# Severe Shoot Trimming and Crop Size as Tools to Modulate Cv. Merlot Berry Composition

**DOI:** 10.3390/plants11243571

**Published:** 2022-12-17

**Authors:** Marijan Bubola, Martina Persic, Sara Rossi, Ena Bestulić, Goran Zdunić, Tomislav Plavša, Sanja Radeka

**Affiliations:** 1 Institute of Agriculture and Tourism, Karla Huguesa 8, 52440 Poreč, Croatia; 2 Polytechnic of Rijeka, Vukovarska ulica 58, 51000 Rijeka, Croatia; 3 Institute for Adriatic Crops and Karst Reclamation, Put Duilova 11, 21000 Split, Croatia

**Keywords:** severe shoot trimming, shoot thinning, Brix, anthocyanins, phenolics

## Abstract

Viticulture production is challenged by climate change and the consequent higher accumulation of carbohydrates in grapevine berries, resulting in high-alcoholic wines. This study investigates the application of severe shoot trimming performed at three different stages and crop size management as tools for the modulation of cv. Merlot berry composition, aimed at reducing the sugar content in the berry. In the first study, the effects of severe shoot trimming carried out at three different phenological stages were studied. In the second study, late severe shoot trimming was combined with two crop sizes and regulated by shoot thinning. The obtained results demonstrated that severe shoot trimming in earlier stages of berry development limited the accumulation of both sugars and anthocyanins as compared to the control treatment. However, when severe shoot trimming was performed at late veraison (at approximately 14 Brix), it decreased only the accumulation of sugars, without affecting the accumulation of anthocyanins. The results of the second study showed that the modification of crop size by shoot thinning significantly affected the measured yield parameters, whereas the effect on Brix and anthocyanins was seasonally dependent. It was concluded that among the studied techniques, severe shoot trimming at late veraison is the most effective way to reduce sugar content in the berry without affecting the accumulation of anthocyanins.

## 1. Introduction

Modern viticulture production has been facing numerous challenges in recent decades because of climate changes and consequently grapevine growers and researchers have to reconsider and adjust vineyard management practices to maintain the desired quality of grapes in traditional winegrowing regions. Among other factors, high temperatures and drought are two parameters that have the greatest impact on grapevine physiological performance in the context of modified growing conditions caused by climate change [1,2]. The most pronounced effect of high temperatures in viticulture is manifested by the earlier onset and shortening of the phenological stages of grapevines [3]. Moreover, as a result of particularly high temperatures during the berry ripening stage, grapes accumulate a high concentration of carbohydrates, resulting in high-alcoholic wines that do not match present consumer preferences and market demands [4]. Furthermore, excess heat between the beginning of veraison and the maturity of the grapes causes a disparity in the sugar/acid ratio and the synthesis of secondary metabolites responsible for the formation of color and aromas, thus directly affecting the final composition of the grapes [5,6]. High temperatures increase evaporation and transpiration, amplifying the effects of drought and water stress in grapevines [1]. Whereas moderate water stress has been proven favorable for obtaining high-quality grapes [7], extreme water stress results in lower yield per vine by affecting bud fertility and berry size [8]. Increasingly frequent heat waves also have a very strong negative impact on grapevine yield, reducing it up to −35% [9].

In recent decades, numerous studies have underlined the need and urgency to implement improved, altered, or new viticultural practices to confront and alleviate the effects of ongoing climate changes [1,10,11,12,13]. Long-term strategies to battle extreme climatic conditions refer to choosing later-ripening varieties, clones, later-ripening and drought-resistant rootstocks, increasing trunk height, and moving vine-growing areas to higher altitudes, whereas short-term strategies include various practices and techniques for crop management [10,13]. There are multiple possibilities to address the high sugar content of grapes through the application of practices that can be carried out in existing vineyards without radical changes. These measures are aimed at the reduction of the assimilation surface, reducing the leaf area to fruit weight ratio, late pruning, the use of shadow nets, earlier harvest, application of anti-transpirant substances, or various combinations of listed practices [6]. Even though the above-mentioned short-term practices efficiently reduce sugar accumulation in grapes, the effect is not uniform among varieties.

Regarding the beneficial effects on berry composition, leaf removal in the cluster zone has been a well-studied vineyard practice in recent decades [14,15,16,17,18]. On the other hand, apical leaf removal or severe shoot trimming with the aim to reduce the assimilation surface and limit sugar accumulation has still not been sufficiently investigated and the available data shows a high divergence in results. When severe shoot trimming is conducted in the pre-bloom stage, it delays ripening, and reduces Brix and anthocyanins [19]. When shoot trimming is carried out after berries set on bush vines, it also delays ripening and lowers sugar content, but has a favorable impact on anthocyanins [20]. If performed at or post-veraison, severe shoot trimming alters the content of Brix and, in most cases, has no negative impact on the content of anthocyanins [21,22,23]. Apart from the effects of the phenological stage on the results of severe shoot trimming, modification of anthocyanins concentration is also cultivar-dependent [24]. In hot Australian climates, leaf plucking of the top two-thirds of shoots apical to the clusters, or shoot trimming at veraison (~14 Brix), did not significantly decrease grape sugar accumulation and its concentration in the berry on the date of harvest [25]. Furthermore, Herrera et al. [26] showed that water stress has a significant impact on severe shoot trimming outcomes, which also partially explains the high variability of results presented in the literature.

Another short-term alternative for reducing the content of total soluble solids in grapes could be the management of crop size, as a high crop size usually decreases the leaf area/yield ratio and reduces the availability of assimilates per unit of grapes [27]. Furthermore, yield formation can be shaped by winter pruning or by thinning the shoots and/or clusters during vegetation [14,28,29]. If the bud load is increased during winter pruning, the limitation of sugar accumulation may not occur due to the absence of a significant reduction in leaf area/yield ratio [27]. On the other hand, the shoot thinning practice is a more reliable technique than bud load management to effectively manipulate the leaf area/yield ratio [30]. To our knowledge, no study has been published to date that has examined the combination of different crop sizes with severe shoot thinning and their possible interactive effects on berry composition.

In order to investigate the possibility to obtain red grapes with reduced sugar content and without negatively affecting the phenolic composition of berries, we tested several canopy management practices on the Merlot variety in a Mediterranean climate, in the Istria wine growing region, Croatia. In the first two-year study (2013 and 2014), we investigated the late source limitation imposed by severe shoot trimming (to canopy height of 65 cm) at 5% or 80% veraison, compared to a treatment where severe shoot trimming at the same canopy height was performed early in the season, when berries were 4 mm in diameter, and to a control treatment where standard trimming was performed at 125 cm canopy height. In the second two-year study (2015 and 2016), we combined two crop sizes (low vs. high crop sizes, obtained by adjusting the number of shoots per vine by shoot thinning) with shoot trimming (severe shoot trimming at 65 cm canopy height at 80% veraison vs. standard shoot trimming at 125 cm canopy height), intending to investigate the relative impact of these two canopy management practices on berry composition, as well as the possible combined effects.

## 2. Materials and Methods

### 2.1. Vineyard Site

The experiment was conducted from 2013 to 2016 at the research vineyard of the Institute of Agriculture and Tourism, located in Poreč (lat. 45°13′20″ N; long. 13°36′00″ E; 15 m asl), in the Istria winegrowing region, Croatia. Merlot (*Vitis vinifera* L.) grapevines (clone 347) grafted on SO4 rootstock (clone 762) were planted in 2006 in chromic luvisol (Terra rossa) soil, on a westerly exposed slope with 5% inclination. Vines were planted with a spacing of 0.8 m within the row and 2.5 m between rows (plant density of 5000 vines/ha) and trained to a vertically shoot-positioned, bilateral spur cordon training system, and pruned to eight to nine spurs containing two buds. Vineyard rows were oriented NNE-SSW, with a declination of 27° from N-S. The vineyard was not irrigated. In order to avoid the formation of dense canopies and to allow for moderate sun exposure of clusters, manual removal of approximately two leaves per shoot in the fruit zone was performed in all treatments at berry setting. Mechanical shoot trimming at a canopy height of 125 cm was performed twice a year for all control (full canopy) treatments, at berry setting (first decade of June) and three weeks thereafter. A third shoot trimming in late July was performed in 2014 caused by excessive lateral growth as a result of high rainfall during the summer. Vines of all severe shoot trimming treatments were initially trimmed in the same way as described here and were additionally and manually trimmed according to the needs of each treatment, as described below.

Meteorological data were provided by the Croatian Meteorological and Hydrological Service. The weather station was located 200 m from the experimental vineyard. Grapevine phenological stages were recorded according to the modified E-L system [31].

### 2.2. Experimental Design

Two different studies were conducted within this research. In the first study conducted during years 2013 and 2014, vines were subjected to severe shoot trimming at three different phenological stages, resulting in the following treatments: (a) severe shoot trimming to 65 cm of the total canopy height, performed when berries were four mm in diameter (SEV-I), at grapevine growth stage 29 according to the modified E-L system (Coombe 1995), (b) severe shoot trimming to 65 cm of the total canopy height at early veraison (SEV-II), at grapevine growth stage 35, when approximately 5% of the berries changed color and the grape juice had approximately 8 Brix, (c) severe shoot trimming to 65 cm of the total canopy height at late veraison (SEV-III), at grapevine growth stage 36, when approximately 80% of the berries changed color and the grape juice had approximately 14 Brix, and (d) untreated control (UC), trimmed to 125 cm of the total canopy height (as described above). The first severe trimming of SEV-I was performed on 17 June 2013 and 9 June 2014. Another additional severe trimming was performed in 2013 (on 5 July) to the same canopy height and two additional severe trimming operations were performed in 2014 (on 1 and 30 July) in order to maintain the same canopy height of 65 cm throughout the season. Treatments SEV-II and SEV-III were severely trimmed only once in each season; SEV-II was subjected to severe trimming on 3 August 2013 and 30 July 2014, whereas SEV-III was subjected to severe trimming on 17 August 2013 and 16 August 2014. In all treatments, shoot thinning was performed at grapevine growth stage 14 according to the modified E-L system [31] in order to leave one shoot per node and to remove stunted shoots. Three adjacent rows were selected to build a randomized complete block design, with each row as a block. Within each row, four sections of two post spaces (12 vines per plot) were tagged and randomly assigned to each treatment. Two post spaces at the beginning of each row were not included in the experiment and were used as buffers. In both seasons, treatments were applied on the same vines. Grapes were manually harvested on 18 September 2013 and 27 September 2014.

In a second study conducted during the years 2015 and 2016, vines were subjected to two crop sizes in combination with two different canopy heights. Two crop sizes were obtained by shoot thinning, which was manually performed at grapevine growth stage 14 according to the modified E-L system [31]. Low crop size treatment (LCS) was obtained by thinning 35% of the shoots, whereas in high crop size treatment (HCS), shoot thinning was not performed. A shoot thinning operation was performed on 29 April 2015 and 20 April 2016. Two canopy heights were obtained by trimming the vines to 125 cm of the total canopy height (high canopy; HC), or by severe shoot trimming to 65 cm of the total canopy height at late veraison (severe shoot trimming; SST), at grapevine growth stage 36, when approximately 80% of the berries changed color and the grape juice had approximately 14 Brix. Severe shoot trimming was performed on 13 August 2015 and 16 August 2016. Treatments were applied in a 2 × 2 factorial design leading to the following combinations of treatments: LCS-HC (low crop size, high canopy), LCS-SST (low crop size, severe shoot trimming), HCS-HC (high crop size, high canopy), and HCS-SST (high crop size, severe shoot trimming). Three adjacent rows were selected to build a randomized complete block design, with each row as a block. Within each row, four sections of two post spaces (12 vines per plot) were tagged and randomly assigned to the four investigated combinations of treatments. Two post spaces at the beginning of each row were not included in the experiment and were used as buffer. In both seasons, treatments were applied on the same vines. Grapes were manually harvested on 17 September 2015 and 22 September 2016.

### 2.3. Leaf Area, Cluster Exposure, and Yield Components

Eight representative shoots per replicate were collected one week before harvest and brought to the laboratory in plastic bags. The primary and lateral leaf area of each sample was determined with a LI-3000 leaf area meter (LI-COR Bioscience, Lincoln, NE, USA). The shoot leaf area was multiplied by shoot number per vine to calculate the whole vine leaf area.

Incident light in the cluster zone, assessed as photosynthetically active radiation (PAR) was determined between 11:00 and 12:00 h on cloudless conditions using a portable QSO-S PAR Photon Flux sensor (Decagon Devices, Pullman, WA, USA), placed vertically and upward near clusters on both sides of the canopy. One hundred clusters per replicate were used for PAR measurements, half of them on the east side of the canopy and the other half on the west side. In the first experiment (in 2013 and 2014), PAR was assessed one day after the execution of severe shoot trimming of the SEV-I treatment and one day after the execution of severe shoot trimming of SEV-III treatment. In the second experiment (in 2015 and 2016), PAR was assessed one day after the execution of severe shoot trimming of the SST treatment.

Point quadrat analysis (PQA) was performed one week before harvest as described by Smart and Robinson [32] in order to determine canopy gaps and leaf layer number in the fruit zone.

Yield and number of clusters per vine were recorded at harvest and the average cluster weight was calculated. The number of clusters per shoot was determined as clusters to shoot ratio (vine basis). Berry number per cluster was calculated from cluster and single berry weight, obtained by a sample of 200 berries.

### 2.4. Berry Sampling and Fruit Composition

During three stages of berry maturation in 2013 and 2014 (at approximately 8, 14, and 18 Brix) and at harvest in all years of study, portions of grape clusters (either wings or cluster tips, including approximately 10 berries) were randomly sampled by scissors to represent different positions in the canopy and within single clusters until approximately the amount of one kg per treatment replicate was reached. A sample from each treatment replicate consisted of approximately 50 portions of clusters, taken from an equivalent number of clusters. Samples were transported to the laboratory within one hour from being harvested. The weight of each sample was taken, and this value added to the yield of the pertinent plot. Berries were cut at the pedicel with scissors to form a sub-sample of 200 berries to be used for average berry weight. At harvest date, these berries were used for total anthocyanins and phenolic determinations. All sets of berries were weighed and immediately stored at −20 °C. The remaining berries (approximately 250) were manually pressed at room temperature, and the juice was used to measure Brix, pH, and titratable acidity (TA).

Brix was determined using a HR200 digital refractometer (APT Instruments, Litchfield, IL, USA), pH was determined using a MP220 pH-meter (Mettler Toledo, Giessen, Germany), and TA (expressed as g/L tartaric acid equivalents) was measured by titration with NaOH 0.1 N as recommended by the International Organization of Vine and Wine [33]. Sugar content per berry was approximated from berry weight and Brix, as reported by Previtali et al. [34]. Total anthocyanins and total phenolic substances in berries were determined as per Iland et al. [35] and expressed either as mg/g of berry fresh weight and mg/berry. All analyses were carried out in duplicate.

### 2.5. Statistical Analysis

In the study of severe shoot trimming at different phenological stages (2013 and 2014), data were processed using GenStat (VSN, Hemel Hempstead, UK; Version 10.2) with a two-way mixed-model ANOVA, where the year was considered as a random factor whereas the treatments were a fixed factor. When differences among treatments were significant, Fisher’s LSD test at *p* ≤ 0.05 was used to separate the means. In the study of severe shoot trimming combined with crop size (2015 and 2016), data were analyzed separately by year using two-way ANOVA in randomized blocks design, for determination of the effects of the two investigated factors (severe shoot trimming and crop size). Values are presented as means over the treatments.

## 3. Results

### 3.1. Meteorological Conditions

**Figure 1:**
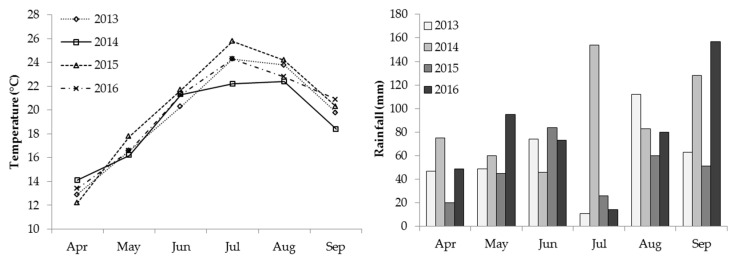
Seasonal patterns of mean air temperature and monthly rainfall in seasons 2013–2016.

The average monthly temperatures and rainfall during the growing seasons (April to September) are shown in Figure 1. Season 2014 was characterized by lower average temperatures from July to September in comparison to other seasons, and higher rainfall in the same period, which was especially high in July. Such conditions had an impact on the slower maturation of grapes, later harvest date, and lower sugar concentration in berries at harvest in 2014. On the other side, season 2015 had slightly higher temperatures from May until August in comparison to other seasons, with lower rainfall throughout the vegetation period, except in June. July was characterized with particularly low rainfall in all seasons, except in 2014.

### 3.2. Study of Severe Shoot Trimming at Different Phenological Stages

As a direct consequence of the removal of the upper part of the canopy, higher values of all leaf area parameters were obtained in the untreated control (UC) as compared to the three investigated severe shoot trimming treatments (Table 1). If severe shoot trimming was performed when berries were four mm in diameter (SEV-I), greater lateral leaf area per primary shoot, total leaf area per shoot, and consequently greater leaf area per vine was obtained as compared to severe shoot trimming performed at early veraison, when approximately 5% of the berries changed color and the grape juice had approximately 8 Brix (SEV-II) or at late veraison, when approximately 80% of the berries changed color and the grape juice had approximately 14 Brix (SEV-III). Such a reaction was a consequence of the regrowth of lateral shoots after conducting severe shoot trimming in SEV-I at this early stage of berry development, when the shoot growth is still active. In fact, the regrowth of lateral shoots in SEV-I was more pronounced than it can be deduced from this data, but the additional shoot trimming was applied on SEV-I to maintain the same shoot height throughout the vegetation period. With this additional shoot trimming, a large part of the developed laterals was removed in both years. Among the three performed treatments of severe shoot trimming, the leaf area/yield ratio was the greatest in SEV-I, and lowest in the SEV-III treatment. Leaf area parameters were unaffected by the season and no interaction between treatment and year was observed.

**Figure 2:**
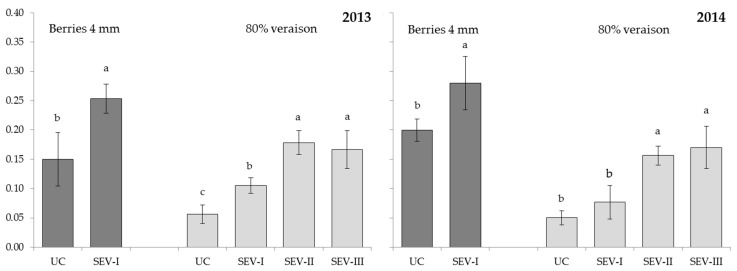
Photosynthetically active radiation (PAR) values in fruit zones for untreated control (UC) and severe shoot trimming treatments applied at different phenological stages (SEV-I, severe shoot trimming when berries were 4 mm in diameter; SEV-II, severe shoot trimming at early veraison; SEV-III, severe shoot trimming at late veraison). Dark grey columns represent PAR values measured one day after the application of SEV-I and light grey columns represent PAR values measured one day after the application of SEV-III. Data were analyzed by one-way ANOVA in randomized blocks design. Different letters identify significantly different means.

After conducting severe shoot trimming at the stage when berries were 4 mm in diameter, significantly higher photosynthetically active radiation (PAR) in the fruit zone was recorded for SEV-I in comparison to UC (Figure 2). In both years of the research, at 80% veraison, there was no significant difference between SEV-II and SEV-III treatments, as both had higher PAR values than UC and SEV-I. In 2013, a significant difference among SEV-I and UC was also noted at 80% veraison, whereas this was not the case in the second year, since vast levels of precipitation promoted the intensive growth of lateral shoots in SEV-I in 2014.

Yield components were not affected by the investigated treatments (Table 2). However, the mean cluster weight and the mean berry weight were higher in season 2014 than in 2013. There was no significant interaction between treatment and year for yield parameters.

The investigated treatments had no effect on titratable acidity, grape juice pH, or total phenolic content of the berries (Table 3), whereas the Brix content and total anthocyanins were significantly affected. Severe shoot trimming when berries were four mm in diameter (SEV-I) and at the onset of veraison (SEV-II) decreased the total anthocyanin content in berries, expressed both as concentration or per berry content, compared to UC. On the other hand, no significant reduction in this regard occurred when the same treatment was applied at 80% veraison (SEV-III).

**Figure 3:**
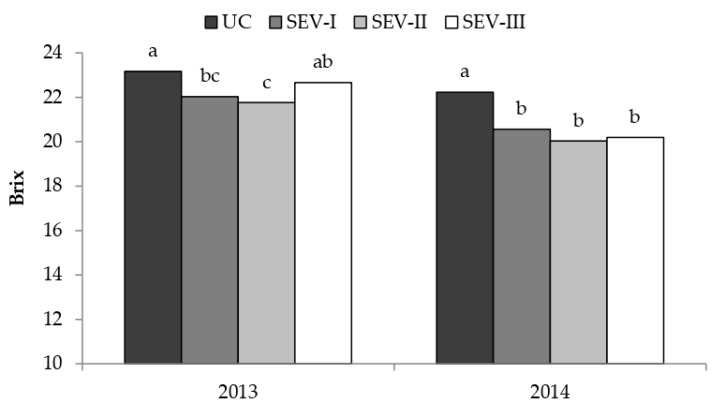
Brix values in berries at harvest date. UC, untreated control; SEV-I, severe shoot trimming when berries were 4 mm in diameter; SEV-II, severe shoot trimming at early veraison; SEV-III, severe shoot trimming at late veraison. Data were analyzed by one-way ANOVA in randomized blocks design. Different letters identify significantly different means.

**Figure 4:**
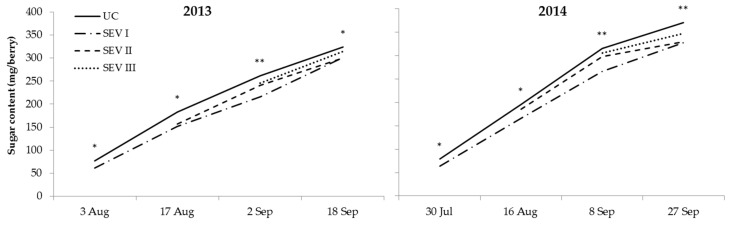
Sugar content in berries (mg/berry) during maturation in seasons 2013 and 2014. UC, untreated control; SEV-I, severe shoot trimming when berries were 4 mm in diameter; SEV-II, severe shoot trimming at early veraison; SEV-III, severe shoot trimming at late veraison. Data were analyzed by one-way ANOVA in randomized blocks design (*, *p* ≤ 0.05; **, *p* ≤ 0.01).

A significant interaction between treatment and year was observed for Brix because treatments SEV-I and SEV-II obtained lower Brix than UC in both years, whereas SEV-III obtained lower Brix than UC only in 2014 (Figure 3). Concerning the sugar content per berry (Figure 4), UC consistently obtained higher values than all the severe trimming treatments throughout the maturation period and in both seasons, whereas SEV-I obtained the lowest values. A greater anthocyanins concentration and per berry content were obtained in season 2013 than in 2014. The concentration of total phenolics was also higher in 2013 than in 2014, whereasa no significant differences were found for the content per berry.

### 3.3. Study of Severe Shoot Trimming Combined with Crop Size

The results of the leaf area and canopy characteristics are shown in Table 4. Severe shoot trimming treatment (SST) had in both years of study a considerable impact on all leaf area components. Lower values of primary shoot leaf area, leaf area of laterals, total leaf area per shoot and per vine, and also lower leaf area/yield ratio was obtained with SST in comparison to the high canopy treatment (HC). On the other hand, low crop size (LCS) significantly increased only total leaf area per shoot in both years, whereas the leaf area of the primary shoot and lateral leaf area per primary shoot increased significantly with LCS only in 2016 and total leaf area per vine was increased with high crop size (HCS) in 2015.

No significant impact of shoot thinning on the leaf area/yield ratio was observed in any season. The results of canopy gaps and leaf layer number did not differ among treatments, whereas PAR in the cluster zone was significantly higher in SST compared to HC treatment in both years of the research.

In both seasons, severe trimming of shoots did not affect any yield parameter, whereas the application of shoot thinning in both seasons reduced the number of shoots per vine, and consequently it reduced the yield per vine and the number of clusters per vine (Table 5). Furthermore, in season 2015, LCS obtained a higher cluster weight and a greater number of clusters per shoot.

The SST treatment had a consistent impact on Brix, which was reduced by this technique in both seasons (Table 6). On the other hand, Brix was significantly reduced by HCS only in 2016. No significant impact of any treatment on the titratable acidity or pH was observed. Although SST had a significant effect on Brix reduction, this technique did not reduce the content of total anthocyanins in berries in any season, which is consistent with the results obtained for SEV-III in a first study conducted during 2013 and 2014. On the other hand, HCS decreased the concentration and per berry content of total anthocyanins in 2016, whereas this effect was not observed in 2015. The content of total phenolics in berries was not affected by the treatments.

## 4. Discussion

### 4.1. Study of Severe Shoot Trimming at Different Phenological Stages

Previous studies reported that removal of leaves on the upper part of the canopy by severe shoot trimming or removing apical leaves on the shoots during the post-veraison period is an effective canopy management practice used to slow the accumulation of sugars in the berries [21,22,23]. Based on this assumption, we tested two different timings of severe shoot trimming during the ripening stage (when approximately 5% or 80% of the berries changed color; at approximately 8 or 14 Brix, respectively) in order to obtain more insight on the impact of the timing of this practice on the final berry composition. Additionally, we have included an early timing of severe shoot trimming, performed when the berries were four millimeters in diameter, at E-L phenological stage 29 according to Coombe et al. [31]. The justification for applying such an early trimming was the possibility of its practical application. If this early trimming technique would be effective in reducing sugars in berries without negatively affecting phenolic composition, this practice would be rational to use in a wide-scale viticulture production because of the ease of its application.

The regrowth of lateral shoots was observed after conducting severe shoot trimming when berries were four mm in diameter (SEV-I), whereas no regrowth of laterals occurred on SEV-II and SEV-III after severe trimming in both seasons. These results for severe shoot trimming in the later phenological stages (SEV-II and SEV-III) confirm a permanent reduction of the leaf area in the current season, as previously reported by Tessarin et al. [36].

The lack of the impact of SEV-II and SEV-III on yield components was expected, as in most studies, yield components were not affected if severe shoot trimming was applied after the onset veraison [37,38,39]. Regarding the SEV-I treatment, we expected a reduction in final berry weight due to a significant decrease in total leaf area per vine in the early stages of berry growth. However, no significant differences were obtained (Table 2.). Several studies on basal leaf removal at berry setting obtained a decrease in the final berry weight [18,40]. In these studies, the lower leaves were removed, which were photosynthetically fully functional at this phenological stage [41], whereas in our study, the removal of upper leaves when berries had four mm in diameter in SEV-I treatment was not limiting for achieving a maximum berry weight. As reported by Kliewer and Dokoozlian [42], for single-canopy training systems, the ideal leaf area/yield ratio for obtaining the largest berry weight and the maximum level of sugar and anthocyanins in the berry ranges from 0.8–1.2 m^2^/kg. In our study, the values of leaf area/yield ratio values remained inside this range for all three severe shoot trimming treatments, therefore giving adequate conditions for uninterrupted berry development.

Even though severe trimming treatments performed in this research did not influence yield components, meteorological conditions during a vegetation period had a significant impact on berry and cluster weight. Greater values of these two variables were obtained in a rainy 2014 season, as high water availability during berry development promotes berry growth [26].

Severe shoot trimming was an effective practice to decrease the rate of sugar accumulation in the berry throughout the maturation stage, and to reduce Brix in the grape juice at harvest. Such a reaction, which was a consequence of late source limitation and the resulting lower assimilate availability to the berries, was confirmed in several other studies on late source limitation, imposed by severe shoot trimming or late leaf removal [21,22,23,43,44,45]. In our study, the most effective treatments in this regard were SEV-I and SEV-II, which reduced the content of Brix compared to UC in both years. The reduction of the content of Brix in SEV-I treatment could be imposed by the significantly lower leaf area/yield ratio than in the control treatment. The SEV-III treatment, where the same practice was performed at late veraison (when 80% of the berries changed color) had a significant impact on the reduction of Brix only in the second year of research. Since a significant interaction of treatment × year was observed, we can assume that severe shoot trimming limits the accumulation of Brix only in years with higher precipitation levels, as in 2014 in our study. These results are in agreement with those presented by Herrera et al. [26] who obtained similar results by investigating the effect of water deficit and severe shoot trimming on the accumulation of sugar in berries, and also with the findings of O’Brien et al. [25], who did not obtain a significant decrease of grape sugar accumulation in the berry by apical leaf removal or by shoot trimming at veraison in a hot Australian climate. If we refer to 2013 as a more average year in terms of temperature and precipitation levels, we can deduce that if apical leaves are removed at late veraison (at approximately 14 Brix), the effect of this practice on the accumulation of Brix is less pronounced than if severe shoot trimming is performed at earlier stages of veraison (at approximately 8 Brix). This arises from the fact that at late veraison, the assimilation area is removed when a substantial portion of the final content of sugar is already accumulated in the berry.

On the other hand, during the rainy season of 2014, all severe shoot trimming treatments obtained a more pronounced decrease in sugars compared to UC than in the previous year. This indicates that a greater leaf area and a greater leaf area/yield ratio are of particular importance when the meteorological conditions during ripening are unfavorable.

The lack of influence of severe shoot trimming on titratable acidity and pH was obtained in several other studies [23,26,36,37], although in some cases, the increase of titratable acidity was observed [45,46], most probably as a consequence of delayed ripening.

Our results imply that if performing severe shoot trimming at an early stage of berry growth (SEV-I) or at the beginning of veraison (SEV-II), a reduced anthocyanin accumulation in berries is obtained. According to Bobeica et al. [24], restricting the assimilation surface, reducing leaf area/yield ratio, and consequently limiting the availability of assimilates at early phenological stages could lead to the prioritizing of primary over the secondary metabolic pathway in berries. However, in our study, this hypothesis was not supported by the results of total phenolic content, which remained unchanged regardless of the phenological stage when severe shoot trimming was performed. The absence of change in the content of total phenolics was also observed in other studies on severe shoot trimming and late apical leaf removal [19,39,43], which indicates a possible shift within the phenylpropanoid pathway in the conditions of limited assimilate availability.

Contrary to the results obtained for SEV-I and SEV-II, no significant reduction of the content of anthocyanins occurred if severe shoot trimming was imposed at 80% veraison (SEV-III), despite the fact that this treatment also had a lower leaf area/yield ratio at harvest than UC. Based on these results, it may be assumed that the timing of performance of severe shoot trimming has a major impact on anthocyanins accumulation in berries, and in order to avoid the decrease of anthocyanins concentration, the best stage to perform this technique is at late veraison, at approximately 14 Brix. Similarly, several other studies obtained the goal of sugar reduction without affecting the accumulation of anthocyanins if late source limitation was performed at late veraison [23,26,37,39], whereas in several cases, a reduction of anthocyanins was obtained if this technique was performed at the beginning veraison [47] or earlier [19,36].

Photosynthetic active radiation in the cluster zone was higher for all the severely trimmed treatments in 2015 and for SEV-II and SEV-III treatment in comparison to UC, as the light entered in the cluster zone to a greater extent also from the upper part of the severely trimmed canopy. A possible impact of light exposure as a factor that could stimulate the accumulation of anthocyanins is questionable in the case of this study, as SEV-I and SEV-II resulted in a reduced concentration and per berry content of anthocyanins than UC, despite the greater light exposure of their clusters. Moreover, it can be assumed that a considerable accumulation of anthocyanins or their precursors in SEV-III treatment occurred prior to the severe trimming of shoots. These results are consistent with recent studies that have shown that solar radiation is not a major factor in anthocyanin synthesis [48,49].

### 4.2. Study of Severe Shoot Trimming Combined with Crop Size

Some interactive effects on berry composition were expected when severe shoot trimming at 80% veraison and its respective control (high canopy; HC) were combined with two different crop sizes per vine. More specifically, we expected that the effects of severe shoot trimming would be more expressed at higher crop sizes. Contrary to our expectations, the results showed that the interaction between these two factors is present only for total leaf area per shoot in the second season, whereas the independent effects of late severe shoot trimming and crop size on total vine leaf area, yield components, and berry composition were observed.

Although it may be expected that shoot thinned LCS treatment would result in more exposed clusters to the sunlight than HCS, no differences in fruit zone microclimate, characterized by canopy gaps, leaf layer number, and PAR were observed between these two treatments. The reason for such outcomes was a more intensive vegetative growth of shoots in LCS treatment compared to HCS (Table 4), resulting in a greater leaf area per shoot in both seasons, a reaction already noted for a differing shoot number per vine [42]. On the other side, a reduction of canopy density following shoot thinning may result if vines have medium to low vigor, where no compensation is obtained in the length of remaining shoots or the number and/or length of laterals [50].

The effects of crop size on fruit composition differed in two investigated years. In 2015, when HCS had 18% greater yield than LCS, no significant impact of crop size on berry composition was achieved. On the other hand, in 2016, when HCS had a 29% greater yield than LCS, the lower content of Brix and total anthocyanins expressed both as concentration and per berry content were observed in HCS. Similar to our study, De Bei et al. [29] found that shoot thinning does not improve berry composition when no considerable differences in yield are obtained with this practice. However, if a considerable difference in crop size among treatments is obtained by shoot thinning or cluster thinning, in most cases, the concurrent reduction of both sugar and anthocyanins is obtained with increased yield [51,52,53,54]. In the context of climate change and the associated problems with high sugar concentration in grape berries in warm seasons [2], the practice of increasing yield may be appropriate to reduce the accumulation of sugar in berries, although it may not be convenient if a high intensity of grape and wine color is desired [55].

## 5. Conclusions

Based on the obtained results, we can conclude that (i) if severe shoot trimming is conducted at early phenological stages of berry development or at the beginning of veraison, it limits the accumulation of sugars and results in a lower concentration of anthocyanins at harvest, (ii) if severe shoot trimming is performed at late veraison (at approximately 14 Brix), it restricts the accumulation of sugar, but does not affect the accumulation of anthocyanins, and (iii) the difference in crop size obtained by shoot thinning does not have a consistent effect on berry composition and does not affect the outcome of severe shoot trimming performed at late veraison.

However, this study also raised additional questions that should be further investigated in future research. Since our results showed seasonal variation, future studies should thoroughly investigate the effect of water stress on the outcomes of late source limitation imposed by severe shoot trimming or late apical leaf removal. Unexpectedly, our results did not confirm the hypothesis of competition between primary and secondary metabolism when assimilates are limited by the removal of a substantial part of photosynthetic active leaf area, as severe shoot trimming performed at earlier stages resulted in lower accumulation of anthocyanins, without affecting the content of total phenolic compounds. These results suggest a possible change in the phenylpropanoid pathway induced by the reduction of assimilate availability on the vine, rather than the competition between primary and secondary metabolism.

The obtained results would also be of practical importance for grapevine producers who may use them to adapt their vineyard management practices according to the specific goals of production and the desired wine style.

## Figures and Tables

**Table 1:** Leaf area of Merlot grapevines subjected to severe shoot trimming at different phenological stages (mean values for 2013 and 2014). UC, untreated control; SEV-I, severe shoot trimming when berries were 4 mm in diameter; SEV-II, severe shoot trimming at early veraison; SEV-III, severe shoot trimming at late veraison.

	Leaf Area of Primary Shoot (m^2^)	Lateral Leaf Area/Primary Shoot (m^2^)	Total Leaf Area/Shoot (m^2^)	Leaf Area/Vine (m^2^)	Leaf Area/Yield (m^2^/kg)
Treatments					
UC	0.163 a ^b^	0.127 a	0.290 a	4.02 a	1.59 a
SEV-I	0.082 b	0.081 b	0.163 b	2.33 b	0.92 b
SEV-II	0.085 b	0.051 c	0.136 c	1.92 c	0.77 bc
SEV-III	0.087 b	0.044 c	0.131 c	1.84 c	0.70 c
*Sign.* ^a^	***	***	***	***	***
Years					
2013	0.100	0.070	0.170	2.47	1.01
2014	0.109	0.073	0.183	2.44	0.94
*Sign.*	ns	ns	ns	ns	ns
Treat × year ^c^	ns	ns	ns	ns	ns

^a^ Data were analyzed using two-way mixed model ANOVA; for significant difference among values, means were separated using Fishers’s least significant difference test. ns, non-significant; ***, *p* ≤ 0.001. ^b^ Different letters identify significantly different means. ^c^ Treatment × year interaction.

**Table 2:** Yield components of Merlot grapevines subjected to severe shoot trimming at different phenological stages (mean values for 2013 and 2014). UC, untreated control; SEV-I, severe shoot trimming when berries were 4 mm in diameter; SEV-II, severe shoot trimming at early veraison; SEV-III, severe shoot trimming at late veraison.

	Yield/Vine (kg)	Clusters/Vine	Cluster Weight (g)	Shoots/Vine	Clusters/Shoot	Berry Weight (g)	Berries/Cluster
Treatments							
UC	2.58	18.9	137	14.0	1.35	1.64	85
SEV-I	2.57	19.0	136	14.2	1.34	1.58	86
SEV-II	2.52	19.2	132	14.3	1.35	1.62	82
SEV-III	2.64	19.3	138	14.1	1.38	1.66	84
*Sign.* ^a^	ns	ns	ns	ns	ns	ns	ns
Years							
2013	2.47	20.0	124	14.6	1.37	1.48	85
2014	2.68	18.2	148	13.7	1.33	1.77	84
*Sign.*	ns	ns	*	ns	ns	**	ns
Treat × year ^b^	ns	ns	ns	ns	ns	ns	ns

^a^ Data were analyzed using two-way mixed model ANOVA; for significant difference among values, means were separated using Fishers’s least significant difference test. ns, non-significant; *, *p* ≤ 0.05; **, *p* ≤ 0.01. ^b^ Treatment × year interaction.

**Table 3:** Berry composition of Merlot grapevines subjected to severe shoot trimming at different phenological stages (mean values for 2013 and 2014). UC, untreated control; SEV-I, severe shoot trimming when berries were 4 mm in diameter; SEV-II, severe shoot trimming at early veraison; SEV-III, severe shoot trimming at late veraison.

	Soluble Solids (Brix)	Titratable Acidity (g/L)	pH	Total Anthocyanins (mg/g)	Total Anthocyanins (mg/Berry)	Total Phenolics (mg/g)	Total Phenolics (mg/Berry)
Treatments							
UC	22.7 a ^b^	6.7	3.36	0.84 a	1.34 a	1.95	3.17
SEV-I	21.3 b	6.8	3.33	0.66 b	1.02 b	1.82	2.85
SEV-II	20.9 b	6.9	3.32	0.69 b	1.10 b	1.84	2.95
SEV-III	21.4 b	6.8	3.31	0.78 a	1.28 a	1.87	3.07
*Sign.* ^a^	**	ns	ns	*	*	ns	ns
Years							
2013	22.4	6.7	3.35	0.90	1.34	2.06	3.04
2014	20.8	6.9	3.31	0.59	1.04	1.68	2.98
*Sign.*	*	ns	ns	**	*	*	ns
Treat × year ^c^	*	ns	ns	ns	ns	ns	ns

^a^ Data were analyzed using two-way mixed model ANOVA; for significant difference among values, means were separated using Fishers’s least significant difference test. ns, non-significant; *, *p* ≤ 0.05; **, *p* ≤ 0.01. ^b^ Different letters identify significantly different means. ^c^ Treatment × year interaction.

**Table 4:** Leaf area and fruit zone characteristics of Merlot grapevines subjected to two different trimming heights (HC, high canopy and SST, severe shoot trimming) and two different crop sizes (LCS, low crop size and HCS, high crop size).

	Season 2015							Season 2016						
	Shoot Trimming			Crop Size			Int. ^b^	Shoot Trimming			Crop Size			Int.
	HC	SST	*Sign.* ^a^	LCS	HCS	*Sign.*	*Sign.*	HC	SST	*Sign*.	LCS	HCS	*Sign.*	*Sign.*
LA of primary shoot (m^2^)	0.204	0.098	***	0.164	0.137	ns	ns	0.191	0.092	***	0.156	0.128	*	ns
Lateral LA/primary shoot (m^2^)	0.101	0.043	***	0.080	0.064	ns	ns	0.136	0.052	***	0.113	0.075	*	ns
Total LA/shoot (m^2^)	0.305	0.141	***	0.244	0.201	*	ns	0.327	0.144	***	0.269	0.203	**	*
% of laterals	33	31	ns	32	33	ns	ns	41	36	ns	41	36	ns	ns
Total LA/vine (m^2^)	4.45	2.06	***	2.99	3.52	*	ns	4.71	2.16	***	3.16	3.72	ns	ns
LA/yield (m^2^/kg)	1.70	0.82	***	1.27	1.25	ns	ns	1.80	0.86	***	1.41	1.25	ns	ns
Canopy gaps (%)	4.7	4.3	ns	5.2	3.8	ns	ns	3.8	3.5	ns	4.2	3.2	ns	ns
Leaf layer number	2.32	2.29	ns	2.13	2.48	ns	ns	2.51	2.44	ns	2.38	2.57	ns	ns
PAR ^c^ (% ambient)	8	15	***	12	11	ns	ns	7	13	**	10	9	ns	ns

^a^ Data were analyzed by two-way ANOVA in randomized blocks design (ns, not significant; *, *p* ≤ 0.05; **, *p* ≤ 0.01; ***, *p* ≤ 0.001). ^b^ Interaction between shoot trimming and crop size.

**Table 5:** Yield components of Merlot grapevines subjected to two different trimming heights (HC, high canopy and SST, severe shoot trimming) and two different crop sizes (LCS, low crop size and HCS, high crop size).

	Season 2015							Season 2016						
	Shoot Trimming			Crop Size			Int. ^b^	Shoot Trimming			Crop Size			Int.
	HC	SST	*Sign.* ^a^	LCS	HCS	*Sign.*	*Sign.*	HC	SST	*Sign*.	LCS	HCS	*Sign.*	*Sign.*
Yield/vine (kg)	2.61	2.53	ns	2.36	2.78	*	ns	2.65	2.59	ns	2.29	2.96	*	ns
Clusters/vine	22.0	22.2	ns	19.4	24.9	**	ns	24.2	23.7	ns	19.8	28.1	***	ns
Cluster weight (kg)	119	115	ns	122	112	*	ns	111	111	ns	116	106	ns	ns
Shoots/vine	14.7	15.1	ns	12.2	17.7	***	ns	15.0	15.2	ns	11.9	18.2	***	ns
Clusters/shoot	1.51	1.5	ns	1.59	1.42	*	ns	1.63	1.57	ns	1.66	1.54	ns	ns
Berry weight	1.49	1.44	ns	1.48	1.45	ns	ns	1.55	1.54	ns	1.59	1.50	ns	ns
Berries/cluster	80	81	ns	83	78	ns	ns	71	72	ns	73	71	ns	ns

^a^ Data were analyzed by two-way ANOVA in randomized blocks design (ns, not significant; *, *p* ≤ 0.05; **, *p* ≤ 0.01; ***, *p* ≤ 0.001). ^b^ Interaction between shoot trimming and crop size.

**Table 6:** Berry composition of Merlot grapevines subjected to two different trimming heights (HC, high canopy and SST, severe shoot trimming) and two different crop sizes (LCS, low crop size and HCS, high crop size).

	Season 2015							Season 2016						
	Shoot Trimming			Crop Size			Int. ^b^	Shoot Trimming			Crop Size			Int.
	HC	SST	*Sign.* ^a^	LCS	HCS	*Sign.*	*Sign.*	HC	SST	*Sign*.	LCS	HCS	*Sign.*	*Sign.*
Soluble solids (Brix)	23.2	21.4	***	22.5	22.1	ns	ns	22.8	21.4	*	22.7	21.5	*	ns
Titratable acidity (g/L)	6.4	6.7	ns	6.5	6.6	ns	ns	6.8	6.8	ns	6.9	6.8	ns	ns
pH	3.37	3.36	ns	3.39	3.34	ns	ns	3.26	3.29	ns	3.28	3.27	ns	ns
Total anthocyanins (mg/g)	0.97	0.91	ns	0.95	0.92	ns	ns	0.83	0.78	ns	0.85	0.76	*	ns
Total anthocyanins (mg/berry)	1.44	1.30	ns	1.41	1.34	ns	ns	1.29	1.22	ns	1.35	1.16	*	ns
Total phenolics (mg/g)	2.20	2.08	ns	2.14	2.15	ns	ns	2.06	1.99	ns	2.08	1.97	ns	ns
Total phenolics (mg/berry)	3.28	3.00	ns	3.17	3.11	ns	ns	3.20	3.08	ns	3.30	2.98	ns	ns

^a^ Data were analyzed by two-way ANOVA in randomized blocks design (ns, not significant; *, *p* ≤ 0.05; ***, *p* ≤ 0.001). ^b^ Interaction between shoot trimming and crop size.

## Data Availability

The raw data supporting the conclusions of this article will be made available by the authors, without undue reservation.
